# The Last Wish

**Published:** 1876-05

**Authors:** 


### THE LAST WISH.

We never fail to feel, when the powerless witness of the last gasp of the dying, *It is worth the effort of a life time to be able to die well.* And then, as well as in the crowded street, or the solitude of our study, we find the inquiry stealing over us, "What would you most wish, when yourself the actor in that last sad scene?" The mental answer has been so prompt, so frequent, so uniform, that it has become stereotyped on the tablet of our memory, and we read it off this beautiful day of departing May, "*That I had been kinder, more indulgent to my fellow strugglers in the great field of life.*"

Reader! How is it with you? Let you and I begin this moment to practise that glorious lesson, and ravishingly sweet will be its memories when we come to die.

